# Response of biochar derives from farm waste on phosphorus sorption and desorption in texturally different soils

**DOI:** 10.1016/j.heliyon.2023.e19356

**Published:** 2023-08-22

**Authors:** Sandeep Sharma, Bharpoor Singh Sekhon, Pritpal Singh, Manzer H. Siddiqui, Mahipal Singh Kesawat

**Affiliations:** a Department of Soil Science, Punjab Agricultural University, Ludhiana, Punjab, India; b Department of Botany and Microbiology, College of Science, King Saud University, Riyadh 11451, Saudi Arabia; c Institute for Molecular Biology and Genetics, School of Biological Sciences, Seoul National University, Seoul, 08826, South Korea

**Keywords:** Biochar, Soil texture, Phosphorus sorption, Desorption, Inceptisol, Langmuir and freundlich isotherms

## Abstract

The information on changes in phosphorus (P) sorption and desorption characteristics and transformations after biochar application to high P fixing soils is still unclear. In the present study, we evaluated the differential response of biochar derives from five different farm waste viz. *Lucaena* sp., *Albbizia* sp., *Mangifera indica*, *Triticum aestivum* and *Oryza sativa* applied at 1 and 3 g kg^−1^ (*w/w*) on P sorption and desorption in three texturally different (silt loam, clay loam and sandy loam) soils. The amount of P sorbed by the clay loam was significantly (*p*<0.05) higher than the silt loam and sandy loam, regardless of added P concentration. The Freundlich isotherms exhibit a better fit (R^2^ = 0.564–0.996 in silt loam, 0.640–0.993 in clay loam and 0.724–0.993 in sandy loam soil) to P sorption data as compared with the Langmuir isotherm. Biochar application significantly decreased the P desorption maxima and desorption constant. The R^2^ values ranged from 0.447 to 0.999 in silt loam, 0.438 to 0.996 in clay loam, 0.545 to 0.989 in sandy loam. *Lucaena* biochar showed highest adsorption maxima, thereby suggesting highest P release, whereas soils treated with *Triticum aestivum* biochar had the lowest adsorption maxima in both clay loam and sandy loam soil. These results indicated that biochar application can significantly enhance P availability; the extent of which is determined by soil texture and type of biochar. The results of present study highlight that biochar application would help increase soil P availability by enhancing fertilizer-P use efficiency associated with decreased P sorption capacity due to increased flush of available-P in soil colloidal complex.

## 1 Introduction

Phosphorus (P) is the most important plant nutrients required for crop production. However, due to the reactive nature of P, the bioavailability of more than 80% of applied P fertilizers can readily become restricted for plant uptake through sorption processes or microbial immobilization [1]. Annually, ∼15 million tons of fertilizer-P is used to meet plant P requirement, but only ∼5–30% of the applied fertilizer-P is absorbed by crops, and the bioavailability of the remaining P is very low due to sorption, precipitation, and microbial immobilization processes (P fixation) that occur in the soil [2]. Phosphorus dynamics in the soils is controlled by the combinations of dissolution-precipitation, sorption-desorption and mineralization-immobilization reactions [3,4] which in turn are influenced by soil properties, of which soil texture is important [5,6]. Therefore, the application of management practices is pressing from the perspective of less P fertilizer application or more P fertilizer use efficiency [7]. Therefore, appropriate evaluation of P availability in a soil is a pre-requisite for providing reliable P fertilizer recommendation and ensuring the long-term sustainable management of agro-ecosystems [[7], [8], [9]]. Application of animal manures [9], crop residues incorporation [7] and planting cover crops are the management practices recommended to increase soil organic carbon (C) storage and improve soil P availability [3,10]. However, due to their low resistance to microbial degradation, effect of these types of organic matter added to the soil decreases or disappears after a relatively short period of time [11]. Over the past decades, there has been a growing interest in biochar utilization as a novel solution for soil amendment [12,13].

Biochar has a significant effect on plant nutrient availability such as P [2,14]. Previous studies have indicated that biochar may act as a slow-release P-fertilizer in soils [15,16]. The application of biochar prepared from different organic sources has been proposed as an option for improving soil fertility, restoring degraded land and to maximize agronomic use efficiency of the applied nutrients which eventually helps enhance the crop productivity [17]. Addition of biochar to the soils has been reported to improve P bioavailability [18,19]. Biochar can affect P bioavailability by altering the soil P sorption and desorption characteristics [20,21]. The mechanisms underlying the altered P availability with biochar application to soil, however, remain poorly understood [22]. The role of biochar in enhancing desorption and therefore, availability of certain nutrient elements has been confirmed by many sorption-desorption studies [[23], [24], [25]]. Biochar application can sorb phosphates (PO_4_^3−^) and reduce P leaching from biochar treated soils [[22], [26]]. The increased P sorption due to increase in exchangeable calcium (Ca^2+^) and magnesium (Mg^2+^) following biochar application has been reported [20,27]. The literature highlights contrasting effects of biochar application did not always increase soil P availability due to wide C: N ratio [12]. Biochar contains a large amount of P and direct release of soluble P may be contributor to enhanced P availability in the soil [4,28,29]. In addition, biochar application reduces soil acidity and subsequently alters P complexation with metals (Al^3+^, Fe^3+^, and Ca^2+^), which is important for estimating P availability by P sorption-desorption reactions in soils [2,13,30]. The sorption of organic molecules on biochar surfaces can reduce their ability to chelate Al^3+^, Fe^3+^, and Ca^2+^ in soil [2,31]. The direct or indirect influence of biochar on the P cycle in soil has not been adequately reported, especially P sorption and desorption after biochar addition to soils [32,33]. Viewing this, overall aim of our study was to study the effect of biochar made from different sources having different biochemical properties on P sorption and desorption in soils differing in texture.

## 2 Materials and methods

### 2.1 Soil and biochar

Three soils differing in textural classes (silt loam, clay loam and sandy loam) were collected from the surface (0–15 cm) soil layer at several sites from the farmers'fields located in Punjab state, north-western India (Table 1). The soil of the experimental field was a *Eutric Cambisols* (IUSS Working Group WRB 2015). The area has a sub-tropical climate, with hot, wet summers and cool dry winters and the temperature varies between 11.6 and 23.9^O^C in winter, 24.8 and 34.3^O^C in summer with mean annual rainfall of ∼769 mm.

**Table 1:** Basic properties of three texturally divergent surface (0–15 cm) soils and elemental composition of different types of biochar used in the study.

Property	Soil types			
	Silt loam	Clay loam	Sandy loam	
pH_1:2_	6.51	7.53	7.2	
E.C._1:2_ (dS m^−1^)	0.168	0.418	0.218	
Soil organic C (g kg^−1^)	0.65	0.59	0.36	
Available-P (kg ha^−1^)	35.8	15.7	24.1	
Available-K (kg ha^−1^)	176	323	105	
	Biochar types			
	N^a^ (%)	C (%)	P (%)	K (%)
Lucaena biochar (*Lucaena* sp.)	0.98	58.9	0.74	1.41
Albbizia biochar (*Albbizia* sp.)	0.66	73.7	0.14	1.52
Mango biochar (*Mangifera indica*)	0.98	77.0	0.32	1.45
Wheat straw biochar (*Triticum aestivum*)	1.72	47.6	0.69	1.72
Rice husk biochar (*Oryza sativa*)	0.59	59.2	1.15	1.50

a Nitrogen (N); carbon (C); phosphorus (P); potassium (K).

Samples were thoroughly mixed, air-dried, passed through a 2-mm sieve and stored at room temperature, before the start of incubation experiment. The physical and chemical properties of the soils are described in detail in Table 1. Five types of biochars were made from different feed stocks viz. *Lucaena* (L), *Albbizia* (A), *Mangifera indica* (M), *Triticum aestivum* (T) and *Oryza sativa* (O) by using portable kiln unit developed by CRIDA, Hyderabad, India [34]. The biochar was ground to pass through a 0.2 mm sieve, and mixed uniformly with the soil. Total N content of biochars was determined by the micro-Kjeldahl method [35]. Total P and K concentrations were determined in triple-acid (HNO_3_:H_2_SO_4_: HClO_4_; 10:3:1) digests using the ammonium molybdate method for P and flame photometry for K [35]. Biochar application rates were set at 1 and 3 g kg^−1^of soil and a control treatment was also kept. At the time of start of incubation experiment, 100 g soil sample was pre-mixed with different biochars as per treatments with three replications. Distilled water was evenly applied to the soil to bring the final moisture to 50% of water holding capacity. After biochar pre-treatment, soils were incubated in plastic containers. These containers were sealed with parafilm and allowed to sit for 15 min for moisture to distribute through the soil. After that, treated containers were shaken to assure adequate aeration in the soils. The sealed containers were placed in an incubator at 25 °C for 60 days and analyzed at 7th, 28th and 60th days after the start of the incubation for P sorption and desorption.

### 2.2 Incubation experiment on P sorption and desorption in biochar amended soil

Five g soil or soil–biochar mixture samples were placed in 50 ml centrifuge tubes and suspended in 25 ml of 0.2 M KCl solution containing 0, 2.5, 5.0, 10.0, 20.0 and 80.0 μg P ml^−1^ added as KH_2_PO_4_. The soil suspension was equilibrated for 48 h with 1 h horizontal shaking twice a day. After the equilibration time was over the samples were centrifuged for 5 min at 5000 rpm in order to obtain the clear supernatant. After filtering, the concentration of P in the filtrate was determined calorimetrically by ascorbic acid method [36] and the amount of P sorbed was calculated by the difference between added and remnant equilibrium solution. P adsorption or sorption data was fitted to Langmuir and Freundlich equations.

#### 2.2.1 Langmuir equation

Cxm=1kb+Cb

Where, C = equilibrium P concentration (μg ml^−1^), x/m = P adsorbed (μg g^−1^ soil), b = Langmuir adsorption maxima (μg g^−1^), k = Langmuir bonding energy constant, that is, the ratio of specific rate constants for adsorption and desorption at equilibrium. The constants ‘k’ and ‘b’ were obtained from the intercept and slope, respectively from the straight-line plot of [C/(x/m)] versus C.

#### 2.2.2 Freundlich equation

$$S={aC}^{1/n}$$

Where, S = amount of P adsorbed (μg g^−1^ soil), C = concentration of P in equilibrium solution (μg ml^−1^), a, n are constants. A linear relationship obtained from the plot of ‘log C’ versus ‘log S’ was used to work out constants ‘a’ and ‘n’ from the intercept and slope, respectively.

### 2.3 Phosphorus desorption studies

Soil samples from the afore mentioned P sorption study were equilibrated with 20 ml of 0.01 M CaCl_2_ solution by shaking them on shaker for 6 h [37]. The soil suspension was centrifuged and filtered. Phosphorus concentration in the solution was determined as described above. The data obtained on P desorption was fitted to the equation.

$$D_{e}/S=1/K_{d}\;D_{m}+D_{e}/D_{m}$$

Where, S is the amount of P adsorbed (μg g^−1^), D_e_ is the amount of P desorbed (μg ml^−1^), D_m_ is desorption maxima (μg g^−1^ soil) and K_d_ is a constant related to the mobility of P in soil.

### 2.4 Statistical analysis

The data were statistically analyzed using analysis of variance (ANOVA) technique in a completely randomized block design using locally developed software ‘CPCS-1’ [38]. One-way analysis of variance was used the least significance difference (LSD) at *p* < 0.05 level of probability to test the significance of treatment means.

## 3 Results

### 3.1 Phosphorus sorption

**Fig. 1:**
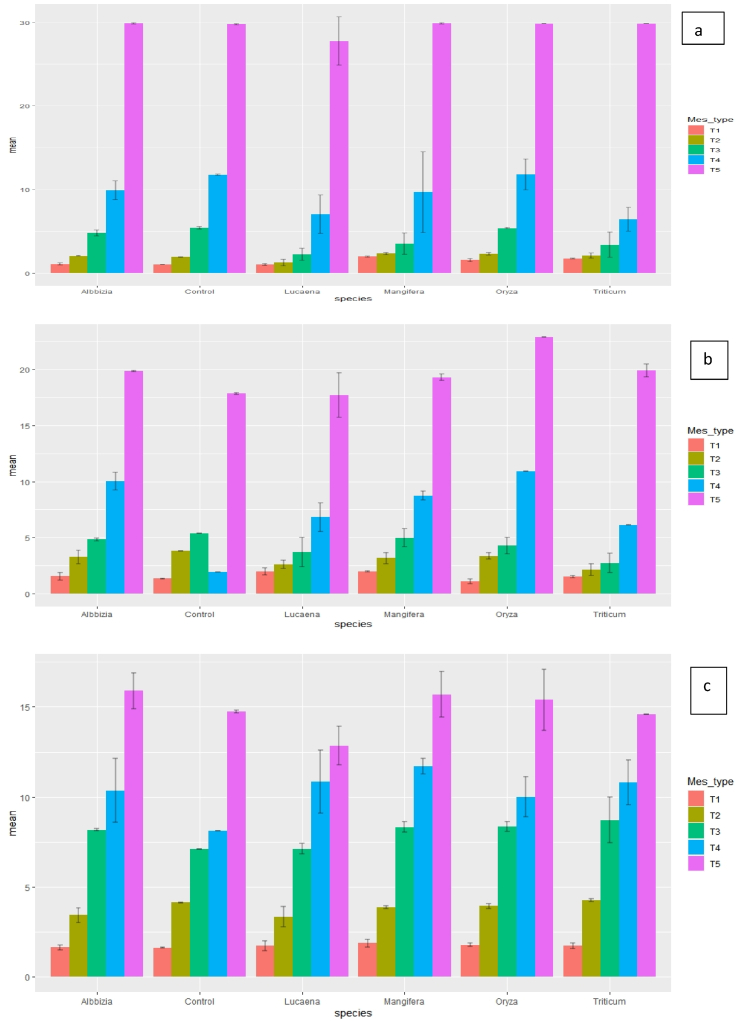
Phosphorus sorption in silty loam soils with different rates of biochar application at different inorganic-P levels after 7, 28 and 60 days of aerobic incubation. (Acronyms: A-7th days; b- 28th days; c – 60th days; T1- 2.5 mg P kg^−1^; T2- 5 mg P kg^−1^; T3- 10 mg P kg^−1^; T4- 20 mg P kg^−1^; T5- 80 mg P kg^−1^).

The P sorption capacity of all the three soils was different and the behavior of P adsorption by different soils at different rates of biochar was not uniform across the whole concentration spectrum. Phosphorus sorption isotherms clearly showed that all the soils had increased affinity for P when different rates of biochar were added to the soil. As rate of biochar addition increased from 1 g kg^−1^ to 3 g kg^−1^, the amount of P sorbed (at 2.5 mg P kg^−1^) decreased with different biochars i.e. *Lucaena* from ∼44.0 to 37.6%; *Mangifera indica* from ∼81.2 to 76.1%; and *Triticum aestivum* from∼ 71.2 to 68.1% in silt loam soil [(Fig. 1 (a-c)]. When biochar was mixed @ 1 g kg^−1^ in silt loam and P addition rate was 2.5 mg kg^−1^, with increase in days of incubation from 28th to 60th days, the P sorption rate increased ∼70.8–76.1% in *Luceana* (L_1_). However, when 80 mg P kg^−1^ was applied the percent adsorption rate decreased (from ∼23.8 to 17.0%) under similar incubation conditions. *Mangifera indica* (3 g kg^−1^; M_3_) showed maximum adsorption percent rate at 7th days (∼76%) and 60th days (80%) of incubation in silt loam soil at 2.5 mg P kg^−1^.

**Fig. 2:**
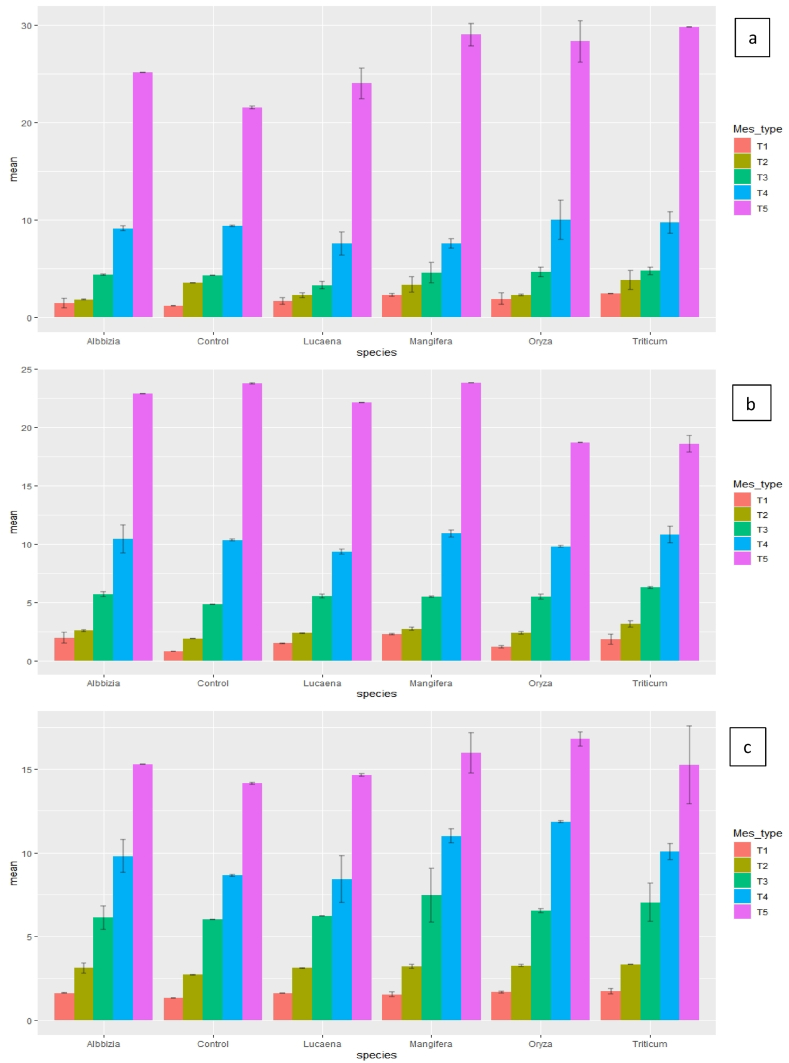
Phosphorus sorption in clay loam soils with different rates of biochar application at different inorganic-P levels after 7, 28 and 60 days of aerobic incubation. (Acronyms: A-7th days; b- 28th days; c – 60th days; T1- 2.5 mg P kg^−1^; T2- 5 mg P kg^−1^; T3- 10 mg P kg^−1^; T4- 20 mg P kg^−1^; T5- 80 mg P kg^−1^).

**Fig. 3:**
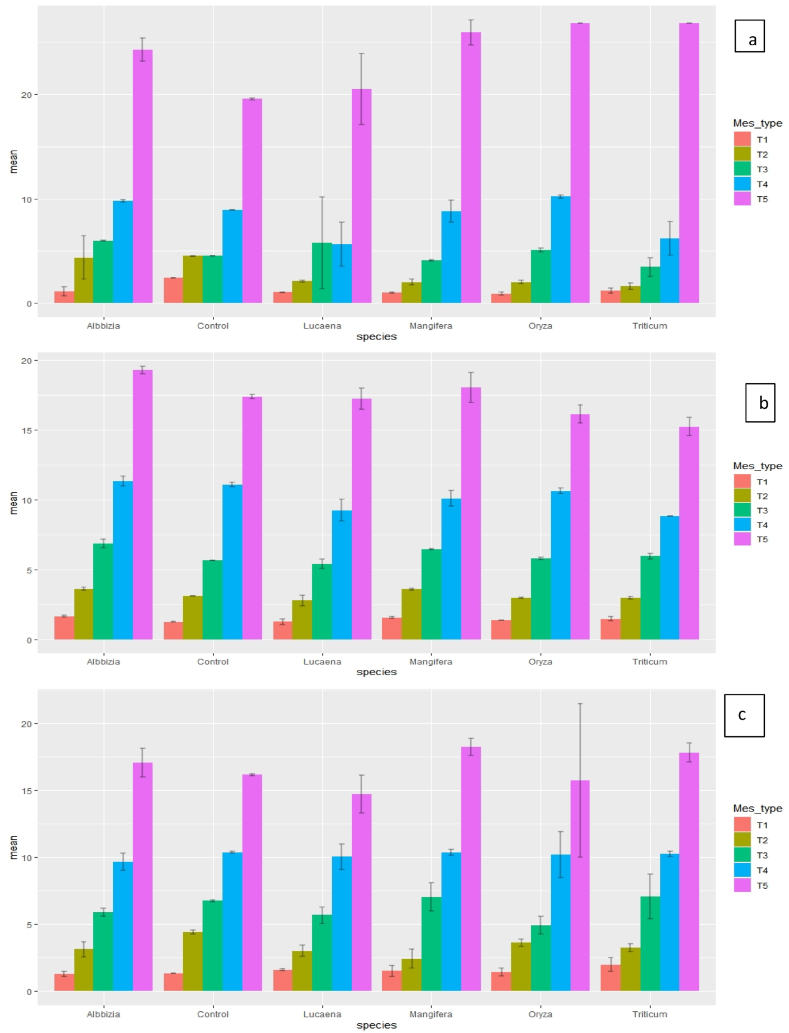
Phosphorus sorption in sandy loam soils with different rates of biochar application at different inorganic-P levels after 7, 28 and 60 days of aerobic incubation. (Acronyms: A-7th days; b- 28th days; c – 60th days; T1- 2.5 mg P kg^−1^; T2- 5 mg P kg^−1^; T3- 10 mg P kg^−1^; T4- 20 mg P kg^−1^; T5- 80 mg P kg^−1^).

In clay loam soil, maximum P sorption (2.46 mg P kg^−1^) occurred when *Triticum aestivum* biochar (T_1_) was applied in the soil followed by *Mangifera indica* (M_1_; 2.21 mg P kg^−1^). In control treatment of clay loam soil (at 2.5 mg P kg^−1^), P adsorption was 46% of added P (1.16 mg P kg^−1^) [(Fig. 2 (a-c)]. However, the percent adsorption rate increased to 98.4 and 72.0 for *Triticum aestivum* biochar under both 7th and 60th days of incubation, respectively. As the days of incubation were increased from 7 to 60 days in *Mangifera indica* biochar (M_1_), sorption rate increased from ∼89.6 to 95.6% (at 2.5 mg P kg^−1^). But when 80 mg P kg^−1^ was applied the percent adsorption rate decreased under similar incubation conditions. In sandy loam soil, maximum P sorption rate occurred with *Albbizia* biochar (A_2_); ∼57.2% and ∼65.2% of added P was sorbed after 7th and 28th days of incubation, respectively [(Fig. 3 (a-c)]. However, in case of 60th day incubation *Mangifera indica* biochar showed maximum adsorption (∼72%) when rate of application was 2.5 mg P kg^−1^. The maximum P sorption rate was ∼23.8% (*Albbizia* under 28th days of incubation) and ∼22.2% (*Mangifera indica* under 60th days of incubation) when both were applied at 1 g kg^−1^ rate and P application rate was 80 mg P kg^−1^. Similarly, in all soils, *Lucaena* biochar showed significantly (*p*<0.05) higher P adsorption maxima at the rate of 1 g kg^−1^, which indicates maximum release of P (Table 2, Table 3, Table 4). Whereas in both clay loam and sandy loam soil, *Triticum aestivum* biochar showed lowest adsorption maxima and in silt loam soil, *Mangifera indica* biochar showed similar results. Among soils, higher P sorption (at each applied P level) in soil occurred in soils having higher clay content. Amount of added P sorbed on soil followed the order of increasing clay content (clay loam > silt loam > sandy loam). Among various treatments, maximum adsorption took place in *Lucaena* at 1 g kg^−1^ rate followed by *Albbizia* at 1 g kg^−1^ rate and maximum release of P took place when biochar was used @ 3 g kg^−1^.

**Table 2:** Langmuir and Freundlich adsorption constants with different rates of biochar application after 7, 28 and 60 days of aerobic incubation on different days of incubation in silt loam soils.

Treatment	After 7th days				After 28th days				After 60th days			
	Langmuir		Freundlich		Langmuir		Freundlich		Langmuir		Freundlich	
	(b) (mg kg^−1^)	(K) (L kg^−1^)	(a) (mg kg^−1^)	(n) (L kg^−1^)	(b) (mg P kg^−1^)	(K) (L kg^−1^)	(a) (mg kg^−1^)	(n) (L kg^−1^)	(b) (mg P kg^−1^)	(K) (L kg^−1^)	(a) (mg kg^−1^)	(n) (L kg^−1^)
Control (CK)	90.9	0.593	3.34	2.45	27.0	0.778	4.15	2.77	6.32	0.750	6.51	4.38
*Lucaena* (L_1_)	71.4	0.120	2.22	1.13	8.69	0.416	2.30	1.92	4.78	0.814	3.56	2.52
*Lucaena* (L_2_)	40.0	0.037	1.74	1.20	1.89	0.440	1.96	1.83	2.99	0.530	2.34	2.15
*Albbizia* (A_1_)	33.3	0.047	1.27	1.01	19.6	0.349	2.53	1.95	4.14	2.670	3.04	2.12
*Albbizia* (A_2_)	31.2	0.015	1.03	1.11	7.14	0.055	1.53	1.46	3.25	0.990	3.12	2.39
*Mangifera* (M_1_)	10.3	0.401	2.27	1.61	5.58	2.610	2.71	2.07	3.58	0.730	4.03	2.55
*Mangifera* (M_1_)	7.6	0.371	1.60	1.68	4.97	0.142	2.30	2.02	2.63	0.650	3.68	2.46
*Triticum* (T_1_)	16.9	0.172	1.56	1.56	12.6	0.448	1.26	1.55	2.09	2.080	5.11	3.87
*Triticum* (T_2_)	14.2	0.177	1.16	1.41	12.8	0.132	1.08	1.60	0.69	0.474	3.63	2.53
*Oryza* (O_1_)	26.3	0.072	1.50	1.33	18.5	0.291	1.52	1.40	3.49	0.534	3.75	2.62
*Oryza* (O_2_)	20.8	0.047	1.59	1.22	10.7	0.103	1.46	1.41	2.21	0.266	3.63	2.55
LSD (*p<*0.05)	7.56	0.15	0.97	0.34	0.39	0.52	0.37	0.42	0.32	0.950	0.30	0.25

**Table 3:** Langmuir and Freundlich adsorption constants with different rates of biochar application after 7, 28 and 60 days of aerobic incubation on different days of incubation in clay loam soils.

Treatment	After 7th days				After 28th days				After 60th days			
	Langmuir		Freundlich		Langmuir		Freundlich		Langmuir		Freundlich	
	(b) (mg kg^−1^)	(K) (L kg^−1^)	(a) (mg kg^−1^)	(n) (L kg^−1^)	(b) (mg P kg^−1^)	(K) (L kg^−1^)	(a) (mg kg^−1^)	(n) (L kg^−1^)	(b) (mg P kg^−1^)	(K) (L kg^−1^)	(a) (mg kg^−1^)	(n) (L kg^−1^)
Control (CK)	66.6	1.140	5.27	3.42	37	3.220	3.79	2.26	11.3	0.220	4.00	2.61
*Lucaena* (L_1_)	31.2	0.065	1.08	1.3	20.8	0.081	1.57	1.46	10	0.190	2.25	1.97
*Lucaena* (L_2_)	0.88	0.350	1.75	1.85	22.7	0.070	1.57	1.46	9.43	0.230	2.15	2.04
*Albbizia* (A_1_)	18.5	0.122	1.18	1.14	16.6	0.100	3.37	1.53	8.19	0.250	2.44	2.04
*Albbizia* (A_2_)	11.2	0.290	1.58	1.52	2.11	0.376	1.79	1.35	11.2	0.160	2.15	1.86
*Mangifera* (M_1_)	3.90	0.314	3.37	2.73	2.77	0.493	3.1	2.12	9.43	0.210	2.25	1.86
*Mangifera* (M_1_)	1.15	0.310	3.37	2.2	1.98	0.361	3.38	2.29	2.93	0.580	3.13	2.24
*Triticum* (T_1_)	0.47	0.228	4.62	3.01	15.6	0.090	2.09	1.72	7.93	0.280	2.47	2.1
*Triticum* (T_2_)	0.38	0.253	1.42	1.36	3.2	0.990	3.35	2.22	4.65	1.140	3.06	2.2
*Oryza* (O_1_)	37.0	0.342	3.2	2.31	28.5	0.628	1.27	1.38	11.9	0.100	2.47	1.87
*Oryza* (O_2_)	66.6	1.140	5.27	3.42	37	3.220	3.79	2.26	11.3	0.220	4.00	2.61
LSD (*p<*0.05)	31.2	0.065	1.08	1.3	20.8	0.081	1.57	1.46	10	0.190	2.25	1.97

**Table 4:** Langmuir and Freundlich adsorption constants with different rates of biochar application after 7, 28 and 60 days of aerobic incubation on different days of incubation in sandy loam soils.

Treatment	After 7th days				After 28th days				After 60th days			
	Langmuir		Freundlich		Langmuir		Freundlich		Langmuir		Freundlich	
	(b) (mg kg^−1^)	(K) (L kg^−1^)	(a) (mg kg^−1^)	(n) (L kg^−1^)	(b) (mg P kg^−1^)	(K) (L kg^−1^)	(a) (mg kg^−1^)	(n) (L kg^−1^)	(b) (mg P kg^−1^)	(K) (L kg^−1^)	(a) (mg kg^−1^)	(n) (L kg^−1^)
Control (CK)	66.6	0.115	1.32	2.09	14.9	0.114	1.84	1.62	2.51	0.437	3.56	2.51
*Lucaena* (L_1_)	50.0	0.040	1.13	1.20	14.3	0.121	1.94	1.73	5.88	0.829	2.51	2.15
*Lucaena* (L_2_)	24.3	0.079	1.25	1.42	28.6	0.064	1.28	1.44	8.4	0.307	2.25	1.97
*Albbizia* (A_1_)	24.3	0.071	1.01	1.18	9.34	0.143	2.74	1.89	9.71	0.252	2.04	1.81
*Albbizia* (A_2_)	25.0	0.060	1.56	1.39	7.81	0.191	2.87	1.89	9.17	0.579	2.03	1.72
*Mangifera* (M_1_)	22.2	0.100	1.39	1.07	7.75	0.266	2.56	1.91	13.3	0.219	1.34	1.42
*Mangifera* (M_1_)	20.0	0.026	1.23	1.10	8.26	0.239	2.49	1.9	5.88	0.769	2.15	1.73
*Triticum* (T_1_)	20.0	0.090	1.47	1.06	10.5	0.223	1.97	1.84	3.62	2.010	3.29	2.26
*Triticum* (T_2_)	30.3	0.108	1.31	1.28	10.4	0.211	2.07	1.88	4.06	1.113	3.54	2.61
*Oryza* (O_1_)	47.6	0.040	1.52	0.98	15.8	0.098	1.93	1.72	10.5	0.180	2.17	1.78
*Oryza* (O_2_)	52.6	0.030	1.09	1.09	9.6	0.204	1.88	1.68	6.7	0.496	2.44	1.91
LSD (*p<*0.05)	10.2	0.02	0.19	0.26	3.3	0.03	0.26	0.28	2.4	0.14	0.78	0.26

The amount of P adsorbed was plotted against its equilibrium concentration to obtain the adsorption isotherm. Langmuir P adsorption maxima and bonding energy constant showed a wide variation among different rates of biochar application. As higher R^2^ (coefficient of determination) values were obtained when the adsorption data was fitted into Freundlich adsorption isotherm (0.564–0.996 in silt loam, 0.640–0.993 in clay loam and 0.724–0.993 in sandy loam) than the Langmuir isotherm equation in all the soils, it indicated that in all the experimental soils, irrespective of biochar treatment, P adsorption followed the Freundlich adsorption model. The value of Freundlich adsorption coefficient was highest in case of clay soil, which is indicative of higher P adsorption capacity followed by the silt loam and sandy loam soil, respectively. The adsorptive capacities of the soils can be described in terms of other constants like 1/n, ‘k’, and ‘b’. In case of clay loam, the highest value of b (66.6 mg P kg^−1^) and k (1.140 L kg^−1^) were found in control, whereas, the lowest b (0.38 mg P kg^−1^) was found in *Triticum aestivum* biochar (3 g kg^−1^) treated soil. The higher P release across all the soils was witnessed when biochar was used @ 3 g kg^−1^ The results indicated that in sandy loam soil having low organic matter, biochar application to soil enhanced adsorption of P but the effect was not considerable. In the clay loam and silt loam soils, having high amount of clay and soil organic C content, large additions of biochar also enhanced adsorption capacity and bonding energy of the soils. The results indicated that biochar application rates for enhancing P availability in variably textured soils need be adjusted accordingly.

### 3.2 Phosphorus desorption

The soils used for P sorption were retained for P desorption. The percentage of desorbed P was higher at higher level of added P in all the soils (Table 5, Table 6, Table 7). The soils which have higher affinity for P adsorption, like silt loam and clay loam, tended to desorb less amount of P, whereas sandy loam desorbed more adsorbed P. The data were fitted to Langmuir desorption isotherm and desorption maxima and desorption constant were calculated. The R^2^ values ranges from 0.447 to 0.999 in silt loam, 0.438–0.996 in clay loam, 0.545–0.989 in sandy loam. The plots of P desorbed vs. P adsorbed indicate differential behavior of different treatments in controlling P availability.

**Table 5:** Phosphorus desorption constants with different rates of biochar application after 7, 28 and 60 days of aerobic incubation on different days of incubation in silt loam soils.

Treatment	After 7th days		After 28th days		After 60th days	
	Desorption maxima (Dm) (mg P kg^−1^)	Desorption constant (kd) (L kg^−1^)	Desorption maxima (Dm) (mg P kg^−1^)	Desorption constant (kd) (L kg^−1^)	Desorption maxima (Dm) (mg P kg^−1^)	Desorption constant (kd) (L kg^−1^)
Control (CK)	0.004	250	0.031	66.6	0.005	166.7
*Lucaena* (L_1_)	0.006	250	0.354	58.8	0.026	50.0
*Lucaena* (L_2_)	0.706	83	0.017	15.3	0.006	142.9
*Albbizia* (A_1_)	0.004	250	0.010	125.0	0.003	166.7
*Albbizia* (A_2_)	0.003	333.3	0.015	76.9	0.006	100.0
*Mangifera* (M_1_)	0.142	45.4	0.015	55.5	0.009	66.7
*Mangifera* (M_1_)	0.110	55.5	0.021	23.8	0.014	52.6
*Triticum* (T_1_)	0.023	90.9	0.012	142.8	0.023	41.7
*Triticum* (T_2_)	0.020	40.0	0.062	62.5	0.051	16.1
*Oryza* (O_1_)	0.005	200.0	0.198	38.4	0.013	66.7
*Oryza* (O_2_)	0.010	142.8	0.009	142.8	0.003	166.7
LSD (*p<*0.05)	0.028	20.3	0.019	23.0	0.011	25.6

**Table 6:** Phosphorus desorption constants with different rates of biochar application after 7, 28 and 60 days of aerobic incubation on different days of incubation in clay loam soils.

Treatment	After 7th days		After 28th days		After 60th days	
	Desorption maxima (Dm) (mg P kg^−1^)	Desorption constant (kd) (L kg^−1^)	Desorption maxima (Dm) (mg P kg^−1^)	Desorption constant (kd) (L kg^−1^)	Desorption maxima (Dm) (mg P kg^−1^)	Desorption constant (kd) (L kg^−1^)
Control (CK)	0.009	200.0	0.008	200.0	0.0026	500.0
*Lucaena* (L_1_)	0.041	100.0	0.007	166.7	0.0047	250.0
*Lucaena* (L_2_)	0.035	21.3	0.043	71.4	0.0066	200.0
*Albbizia* (A_1_)	0.004	500.0	0.009	200.0	0.0059	250.0
*Albbizia* (A_2_)	0.062	38.5	0.042	25.0	0.0211	111.1
*Mangifera* (M_1_)	0.020	8.5	0.068	34.5	0.0037	333.3
*Mangifera* (M_1_)	0.045	27.8	0.043	21.7	0.016	90.9
*Triticum* (T_1_)	0.020	6.6	0.008	166.7	0.0054	250.0
*Triticum* (T_2_)	0.012	1.4	0.033	20.0	0.0084	111.1
*Oryza* (O_1_)	0.077	25.0	0.005	333.3	0.0021	500.0
*Oryza* (O_2_)	0.012	200.0	0.004	333.3	0.0043	333.3
LSD (*p<*0.05)	0.018	25.8	0.013	30.9	0.04	56.1

**Table 7:** Phosphorus desorption constants with different rates of biochar application after 7, 28 and 60 days of aerobic incubation on different days of incubation in sandy loam soils.

Treatment	After 7th days		After 28th days		After 60th days	
	Desorption maxima (Dm) (mg P kg^−1^)	Desorption constant (kd) (L kg^−1^)	Desorption maxima (Dm) (mg P kg^−1^)	Desorption constant (kd) (L kg^−1^)	Desorption maxima (Dm) (mg P kg^−1^)	Desorption constant (kd) (L kg^−1^)
Control (CK)	0.053	16.9	0.014	90.9	0.037	71.4
*Lucaena* (L_1_)	0.006	250.0	0.01	142.8	0.02	111.1
*Lucaena* (L_2_)	0.038	111.1	0.011	166.6	0.017	111.1
*Albbizia* (A_1_)	0.01	142.8	0.009	125.0	0.006	166.6
*Albbizia* (A_2_)	0.006	200.0	0.0058	166.6	0.008	142.8
*Mangifera* (M_1_)	0.0038	333.3	0.011	125.0	0.003	333.3
*Mangifera* (M_1_)	0.101	90.9	0.0092	142.0	0.003	250.0
*Triticum* (T_1_)	0.0042	250.0	0.0048	200.0	0.014	71.40
*Triticum* (T_2_)	0.027	111.1	0.0087	125.0	0.020	13.50
*Oryza* (O_1_)	0.0021	500.0	0.0033	333.3	0.008	166.6
*Oryza* (O_2_)	0.0019	500.0	0.003	333.3	0.0059	166.6
LSD (*p<*0.05)	0.011	62.9	NS	50.0	0.013	58.2

The desorption maxima (Dm) serves as an index of adsorbed P. More the Dm value lesser is the potential of soil to release P. The desorption constant (Kd) measures the resistance to P desorbed, higher the value of Kd lower the potential of soil to release adsorbed P. Desorption maxima was maximum in control treatment and its values decreased under biochar treatment. At the start of incubation, in silt loam soil, the maximum desorption (333.3 L kg^−1^) was in control and as the rate of biochar increased from 1 g kg^−1^ to 3 g kg^−1^, there was decrease in desorption constant showing release of P in the soil. Similar trend was found in clay loam and sandy loam soil in all the treated soils. The results revealed that biochar incorporation lowered the values of both desorption maxima and desorption constant, thereby indicating a more favorable effect on P availability in the loamy sand soil than in clay loam and sandy loam soil. Maximum adsorption was found in clay loam and maximum desorption was found in sandy loam soil.

## 4 Discussion

The behavior P adsorption and release in all soil varied widely both with type of biochar and soil texture. Phosphorus sorption by various biochar amended soils seems to be controlled by anion exchange between anions of P in solution and the oxygenated functional groups on surface of biochar [12,39]. As the amount of added P increased from 2.5 to 80 mg P kg^−1^, the availability of adsorbed P was significantly different among various biochar types. At low P concentration in solution (2.5 mg P kg^−1^), the availability of P was low due to formation of bidentate complexes by anions of P at high energy sorption sites of surface of sorbent [39]. However, at high solution P concentrations (80 mg P kg^−1^), the arrangement of exchangeable monodentate complexes along with bidentate complexes by P ions on biochar surface may contribute to labile pool of P [40]. As the incubation period increased from 0 to 60 days, there was decline in adsorption constants showing more release of P in the soils. Deluca et al. [19] reported that biochar contains a large amount of P, which may be playing a significant role in enhancing P availability. Biochars, particularly hydrophobic or charged (negatively/positively), can adsorb organic molecules (such as complex proteins, phenolic acids and carbohydrates) that can act as chelates of metal ions that otherwise precipitate P. In other words, organo-biochar or organo-mineral-biochar complexes are created over time, increasing P solubility and availability [41]. Biochar can alter soil P chemistry by interaction with soil organic and mineral components and by changing P chemical forms and the soil's P sorption and desorption capacity [20,42]. In fact, organic amendments can provide nutrients and favorable conditions for the microbes to mineralize and solubilize organic and inorganic forms of P [43]. According to Singh et al. [44] during organic matter decomposition, P assimilated by microorganisms becomes immobilized in the form of nucleoproteins, lipids, and other organic compounds. Higher adsorption of P observed in clay loam and silt loam may be attributed to the high clay content and cation exchange capacity (C.E.C.) of the soils. Wang et al. [45] also reported that fine fraction (silt + clay particles) had high specific surface area and high P sorption capacity, while the sandy loam had the lowest sorption capacity for P due to its low sorption capacity. Wisawapipat et al. [46] reported that sorbed P was more readily available in soil solution where soils were low in clay content. This agreed with Sonnie et al. [47] who reported that decreased P sorption in coarse textured soils resulted from the increase in soil pH and from competition for P sorption sites in biochar amended soils.

Soil clay content has significant influence on soil P sorption indices such as Langmuir constants [48]. The P adsorption isotherms suggested that though the sorption of P increased with its increasing concentration in the equilibrium solution, yet the percentage of sorbed P decreased due to increase in the ratio of adsorbate to adsorbent. Xu et al. [20] reported that P sorption data of biochar treated soil sample can be described by Langmuir equation and P sorption increased with increasing P concentration in the soil. The increase in the values of Langmuir sorption constants with increasing clay and biochar content leads to increase in the P adsorption and decreases the equilibrium P concentration in soil solution [49,50]. Regardless of the treatments (Biochar type and application rates), the amount of P desorbed increased with increasing amount of adsorbed P [51,52]. The data showed that the soil that desorbed a higher percentage of applied P during adsorption, tended to desorb a lower quantity of adsorbed P in desorption step and *vice-versa*. The data indicate that desorption is a slow process and it may be behaving differently from adsorption in soil, which indicates that sorption and desorption of P were inversely related and the soils that adsorb P most readily, release it the least in the soil solution [53,54]. The rate of biochar had a significant effect on P release in the soil. More release of P was found as the rate of biochar increased from 1 to 3 g kg^−1^. Xu et al. [20] reported that a lower rate of biochar amendment fixed the adsorbed P more strongly, but did not easily desorb it. Desorption was found to be the lowest with clay loam soil compared with silt loam soil and sandy loam soil. Decrease in P desorption with clay may be due to their greater affinity to fix P, as the main phosphate sorbing soil surfaces are those of edges of silicate clays [55].

## 5 Conclusion

The soils amended with biochar not only varied in their capacity to adsorb P, but also in the energy with which they retain P depending upon soil texture. Adsorption of P followed Freundlich adsorption model. Adsorption capacities (K values) worked out by Freundlich equation had significant correlation with clay content. Langmuir adsorption isotherm was linear at all the concentrations in all the soils. The fine textured soils had higher desorption maxima and more mobility of adsorbed P compared with coarse textured soils. Coarse textured soils showed higher P adsorption with lower level of biochar whereas fine texture soils behaved better with higher rates. Biochar applications hold a great promise in enhancing P availability in soils due to yielding of phosphate ions to the competition from organic anions and due to the inherent P content of biochar materials.

## Author contribution statement

Sandeep Sharma; Bharpoor Singh Sekhon; Pritpal Singh: Conceived and designed the experiments; Performed the experiments; Analysed and interpreted the data; Contributed reagents, materials, analysis tools or data; Wrote the paper. Manzer H. Siddiqui; Mahipal Singh: Contributed reagents, materials, analysis tools or data.

## Data availability statement

Data included in article/supp. material/referenced in article.

## Funding

This work was funded by the Researchers Supporting Project number (RSP2023R347), 10.13039/501100002383King Saud University, Riyadh, Saudi Arabia.

## Declaration of competing interest

The authors declare no competing interests.
